# Why Nanoscience Needs Standardized Protocols—And How to Get There

**DOI:** 10.1021/acsnanoscienceau.5c00028

**Published:** 2025-05-14

**Authors:** Marek Grzelczak

**Affiliations:** 202635 Centro de Física de Materiales (CFM-MPC), CSIC-UPV/EHU, Paseo Manuel de Lardizabal 5, 20018 Donostia-Sebastián, Spain

**Keywords:** Nanoscience, FAIR principle, pipelilne, open science, data management

## Abstract

Nanoscience is a relatively young research field that has been built on the shoulders of consolidated areas ranging from solid-state physics to biology. Its interdisciplinary nature imposes the flow of heterogeneous data from various domains of predefined conventions that ultimately prevents workflow standardization, raising the possibility of further fragmentation and compromising the reproducibility. This is the time to establish good practices for experimental nanoscientists. This work proposes a set of simple rules that can facilitate data management and improve their reusability. Implementing the proposed protocol can have high initial cognitive costs but can also save energy and time in the long term. By adopting these practices, researchers can ensure the reusability of their data early in a project and accelerate the writing process.

## Graphical abstract

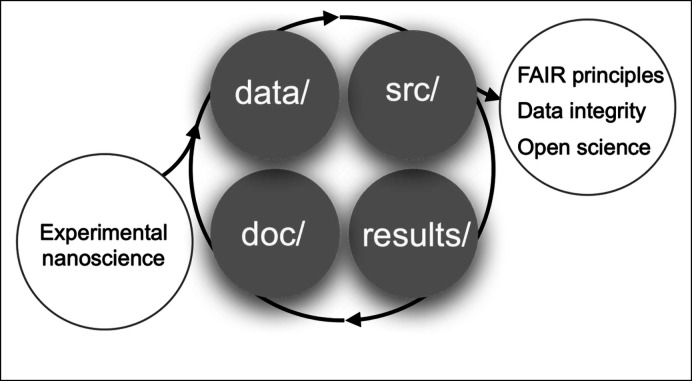

## Introduction

In the year 1900, Henry Ford revolutionized the manufacturing process with a simple yet groundbreaking solution. By implementing a protocol where workers remained stationary while the production line set vehicles in motion, productivity rates soared. The Ford model has become a staple across many manufacturing domains. There is a sector awaiting its own transformative innovation akin to Ford’s invention, the knowledge sector.^1^

After the emergence of the knowledge sector in the late 1960s, a ’hands-off’ approach prevailed, as trained and specialized personnel were expected to develop their work protocols to suit their irregular work style.^2^ But the revolution had never happened. Even worse, with the ascendancy of digital capitalism propelled by the attention economy, knowledge workers (we) found themselves vulnerable to an inundation of asynchronous messaging systems and an overwhelming array of AI-aided tools designed to enhance productivity. The knowledge sector, particularly within academia, needs standardized work protocols to align with FAIR principles (findability, accessibility, interoperability, and reusability)^3^ imposed by funding entities.

But what kind of standardized protocols can we design and implement? Notably, software developers possess valuable insights in this regard. As shown by Wilson et al.,^4^ software developers succeeded in implementing practices that promote efficiency and standardization within their workflow (e.g., Agile framework). Beyond conventional measures, software developers also integrated disruptive mechanisms for team collaboration, such as Scrum^5^ method or extreme programming.^6^ More recently, several groups have proposed automation tools for writing research papers.^7^ These tools, indeed, can enhance the organizational structure, communication, and dissemination of research outcomes within the experimental research community.

By following the software developers’ practices,^4^ I propose in this paper a set of standardized processes, including directory tree structuring, file naming, data processing workflow, and guidance on the completion of the smallest units of publishable information in the form of weekly or biweekly reports. The text is addressed primarily to early-stage researchers, who are the driving force of nanoscience and who produce and manage the data to generate publishable results that are expected to be aligned with open science policies.

## Publon—Quantum of Publication

Knowledge has a pyramidal shape.^8^ At the very bottom, a broad layer of data resides, followed by information and knowledge. On top sits wisdom. Such a hierarchy of data-information-knowledge-wisdom is intrinsic to the scientific method. In practice, the knowledge sector knows that the pyramid is less structured (see ref 9) and that, nowadays, the data layer is barely manageable, especially by newcomers.^10^ It is hard to grasp meaningful information from a flood of data unless we are armed with community-accepted protocols and the right digital tools.

In his book titled *A PhD is not enough*, Peter J. Feibelman introduced the term publon.^11^ Publon is kernel information produced in a time window while conducting research. Publon is a quantum of publication—the smallest unit of publishable information that can eventually become a paragraph, section, or figure in a research article (Figure 1). Therefore, publon is a report that constitutes a sequence of logically organized thoughts. The generation of a publon requires six steps (Figure 1): (1) formulation of the question, (2) literature search, (3) formulation of hypothesis, (4) experimental or computational work, (5) data analysis, and (6) reporting. In the last section, we will discuss the workflow in more detail. The true reward of publon approach comes from consistency and machine-like precision, as acquiring knowledge requires a systematic approach. Implementation a publon is very much the same as implementing a new habit in professional life. Its repetition leads to the formation of the so-called habit cycle.^12^ Over time, a whole network of interconnected publons emerges, forming a large tree bearing new ideas that can be crystallized if managed by the right productivity-based software (Obsidian, Roam, Logseq) or ChatGPT operating offline.^13^

**Figure 1:**
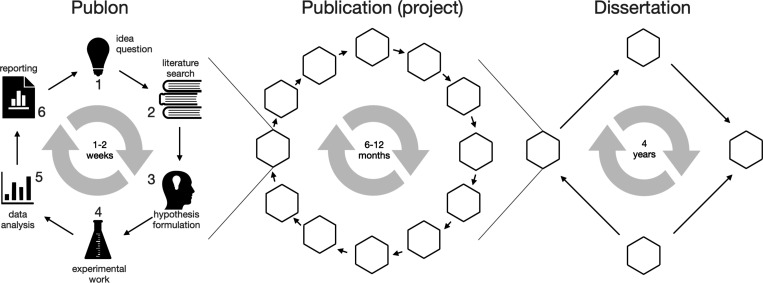
Hierachical structure of scientific method, with the publon being the smallest unit of publishable knowledge.

Consistent generation of publons renders scientific publication; a completed project (Figure 1). By project, I mean a collection of tasks executed within a defined deadline; a project without a deadline is a dream. The collection of publications gives rise to a dissertation. Beyond the thesis, a collection of dissertations and papers comprises a whole research line. But, the constantly accelerating pace of science communication has the potential to make a publon a standalone publication. Recently, the Digital Discovery journal launched a new type of scientific document—Commit—that aims to report improvements on one’s own—or other’s—work.^14^ Thus, a publon is a documented set of results, whether negative or positive, offering novelty that can become a publication, serve as a part of a running paper, be a claim in a patent application, be the foundation of a whole PhD program, or provide preliminary results in a research proposal. In a way, publon resembles the sprint method, a commonly used approach in many industries, where a team focuses entirely on answering a given question to deliver a prototype (evaluate the hypothesis) in a short time frame.^15^

## Folder Structure

All research projects generate large amounts of data. Independent of discipline, a project is structured in nested directories organized to facilitate access to a given file. (Note that the words “folder” and “directory” have the same meaning here.) Everyone runs a custom-made folder system that allows for finding the desired information. An efficient system is user-agnostic; others must access a particular file. No one wants to spend more than 5 min looking for a file created by another researcher five years earlier. A universal data structure ensures the findability and accessibility of all produced data.

A project is a set of logically organized tasks to meet a specific goal within a given deadline. The physical manifestation of a project is a set of hierarchically organized dictionaries. Like publon, the folder structure should be modular and capable of hierarchical organization, a blueprint for an individual research article.

The highest level of our folder system comprises four primary dictionaries: ‘data’, ‘doc’, ‘results’, and ‘src’ (Figure 2). As its name indicates, the ‘data’ contains all raw data. The ‘doc’ contains reports and drafts, all the documents generated during project execution. The ‘results’ folder is for plots and figures to generate reports and papers, while the ‘src’ folder is to store working scripts to analyze raw data and generate processed data, plots, and figures. The rule of thumb is to maintain the location of all files and scripts in the respective folders to make the search more intuitive and to avoid path corruption while executing scripts.

**Figure 2:**
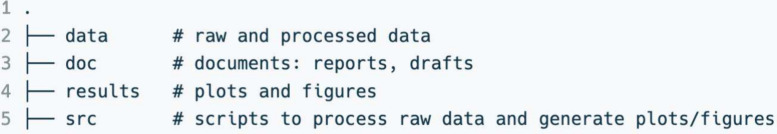
Top-level folder structure comprising four main folders used to organize the whole project.

Moving down the directory tree, one finds project-specific names (Figure 3). For example, two folders in the ‘data‘ contain raw data (for file naming rules, see below). The ‘doc’ folder is the home for drafts and reports. The main scripts go to the ‘src’ folder, while auxiliary scripts stay in the ‘misc’ folder. Template of the project folder can be accessed from Dropbox,^16^ GiHub,^17^ or upon request.

**Figure 3:**
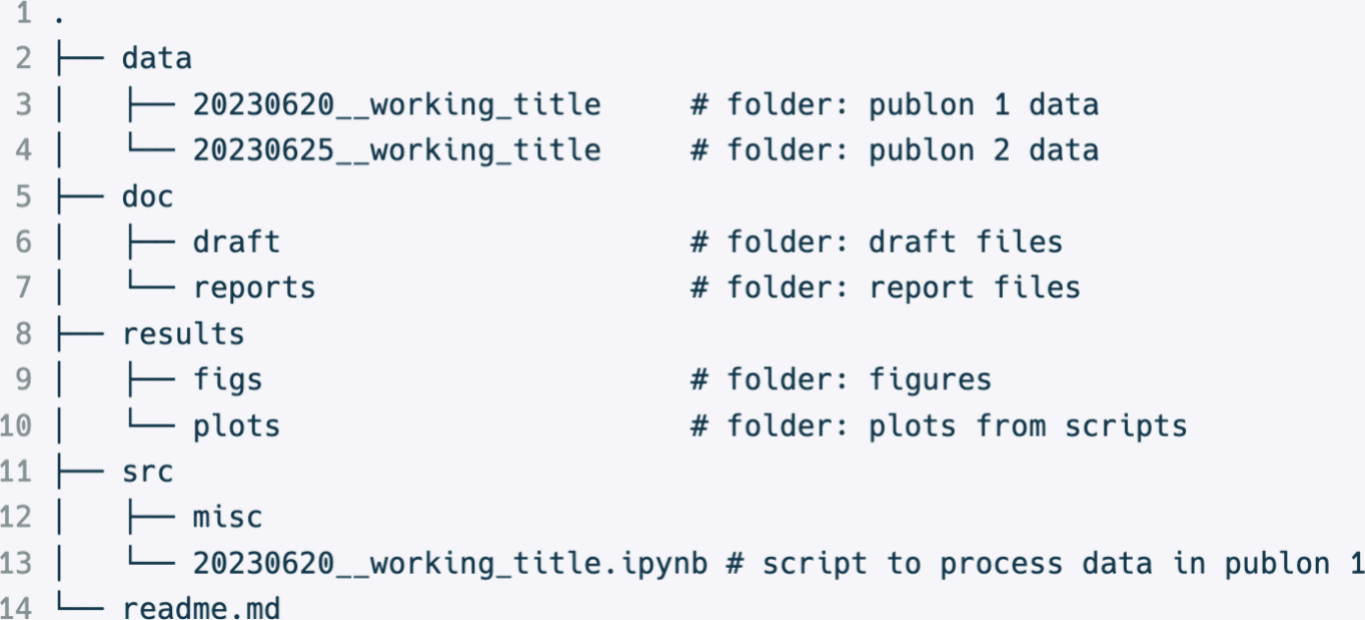
Second-level folder structure. The ‘data’ folder contains folders with raw data. These folders’ names contain creation date and working title. The “doc” folder contains ‘draft’ and ‘reports’ folders. The content of the ‘reports’ folder includes ingredients of the ‘draft’ folder. The ‘results’ contains ‘figs’ and ‘plots’ folders. The ‘plots’ folder is the home for individual files containing plots, while ‘figs’ are the final version of figures composed of several plots. Figures will be used in the final publication. The ‘src’ folder contains all scripts needed to analyze and plot data. The name of the script file should be the same as the folder containing the data used to feed the script.

This simple folder system is compatible with widely spread systems not related to nanoscience, such as, for example, PARA system^18^ by Tiago Forte (PARA stands for Project, Area, Resource, Archive). Typically, I store the folders of running projects into the Project folder, and once completed, I move them to Archive.

## File Naming System

The FAIR principle requires metadata to be readable by humans and machines. Proper file naming is the first step in meeting this requirement, especially at an early stage of data acquisition. Typically, data gathered under a given publon are grouped in the same folder, ‘20230620__working_title’ in Figure 3—lines 3 and 4. The numbers correspond to the date the directory was created, being thus an ID of a publon. Similarly, the ‘working_title’ is the name and ID of the current project (Figure 4, lines 4–6). Notice the convention of a double underscore symbol (‘__’) separating the date and working title. This facilitates automated data filtering for later uploads to repositories. Usually, we do not construct plots or figures from an individual file but rather from tens or even thousands of files, which often require data processing. Therefore, raw data are grouped from different sources (experimental techniques) within one publon folder (Figure 4, lines 4–7). Let’s us contemplate an example. One decides to study the effect of the concentration of a given chemical on the properties of the nanomaterial. The collected data from various instruments—spectroscopy, microscopy, digital images—are placed in corresponding folders (Figure 4, lines 4–6). Although the data come from different techniques and with varying time stamps, they belong to the current publon; that is, they are placed in the folder under the name of the given working title and creation date. The folder is declared closed once the publon is delivered (report). The acquisition of a new raw data set begins in a folder of a different date (publonID) but with the same working title (project ID).

**Figure 4:**
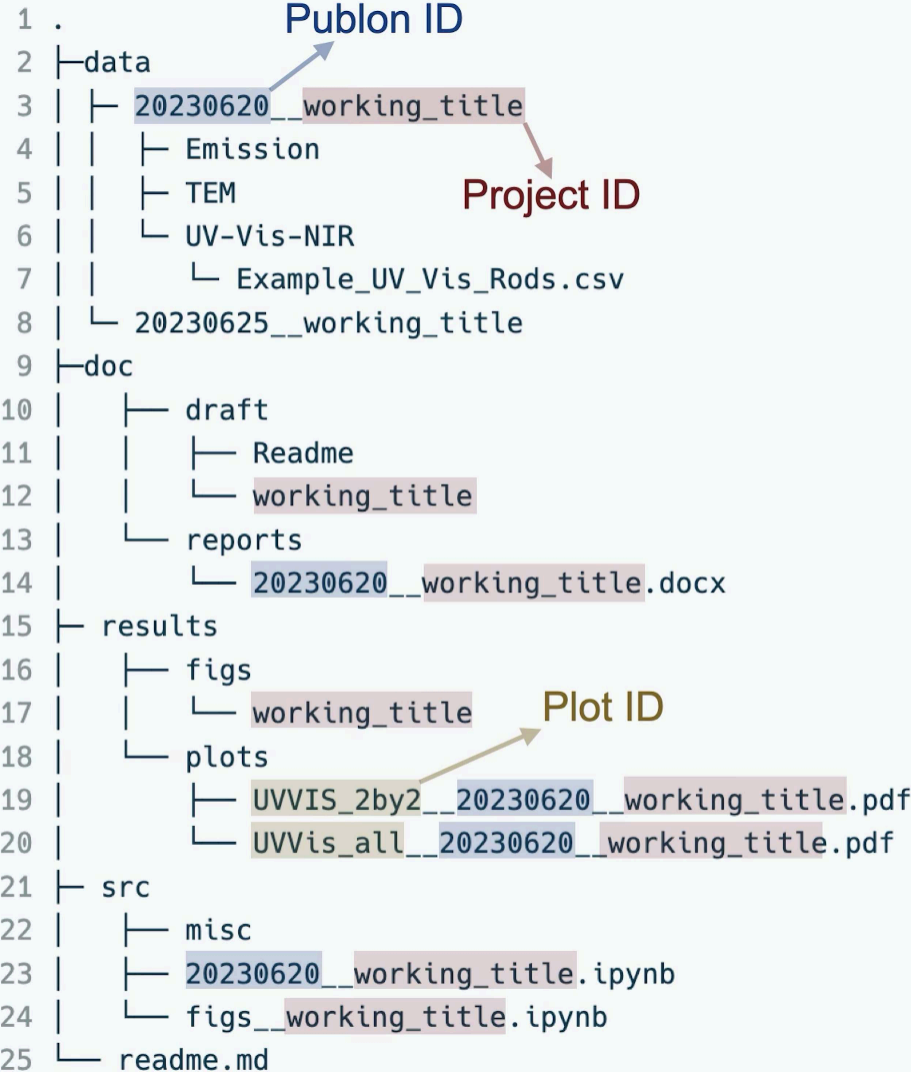
Third-level folder structure with highlighted names of the folders and files. The ‘working_title’ is the ID of the project, and the selected name is shared across files and folders. It is recommended to not change the name. The ‘creation date’ is placed when new folder is created, and it is the ID of the current publon. In the plots folder, the file names should contain the date, working title, and ID of each file.

The consistency of file naming is critical to ensure back-referencing of data. The selected name for a project (‘working_title’) remains unchanged throughout the project’s lifetime. It is essential as the name will be used to name files in the ‘src’ or ‘plot’ folders. Put differently, the name conveys information shared between the raw data, the script processing the data, and the resulting plots. For example, the Jupyter notebook file ‘20230620__working_title.ipynb’ in Figure 4 (line 23) has a name matching the name of the folder data ‘20230620__working_title’. Thus, the script does not need to be launched to locate the source data. Similarly, the folder with the name ‘results/plots’ (Figure 4, lines 19 and 20) contains a couple of pdf files (e.g., ‘UVVIS_2by2__20230620__working_title.pdf’). These plots were generated by the script file named with the date and working title, which is located in the ‘src’ folder. The first term of the file (e.g., ‘UVVIS_2by2’) is the ID of the plot file. Consistent naming of files and folders accelerates access to generated data and facilitates reusability and findability.

## Workflow: From Publon to Publication

As stated above, the generation of a publon involves six steps. In this section, we discuss its practical implementation along with the timeframe needed to accomplish each step.

### Idea

Typically, the idea or driving question of a publon emerges while analyzing data from the previous publon or discussing the report (required time: a few minutes).

### Literature Search

Before going to the laboratory, a literature search is needed to find relevant prior works that potentially answer the formulated question. As of this writing, the available digital tools such as Google Scholar, Sci Finder, and Perplexity allow for fast screening of relevant references. This step should never be omitted. As Paul Mulvaney advises, “Remember that six months in the lab can always save you an hour in the library” (required time: 2–3 h).

### Hypothesis Formulation

At this step, the working hypothesis is formulated, all experiments planned, and all possible outcomes and problems visualized. The limitations of the experimental approach are defined (required time: 60 min).

### Data Acquisition

This is the time for running experiments and acquiring the data. A new folder in ‘data’ is created with a name containing publon and project ID (Figure 5, left - step 1). Data are grouped by experimental/computational technique. The naming of raw data in each folder should be robust and future-proof; an ASCII format (txt, csv) is recommended (required time: 3–4 days).

**Figure 5:**
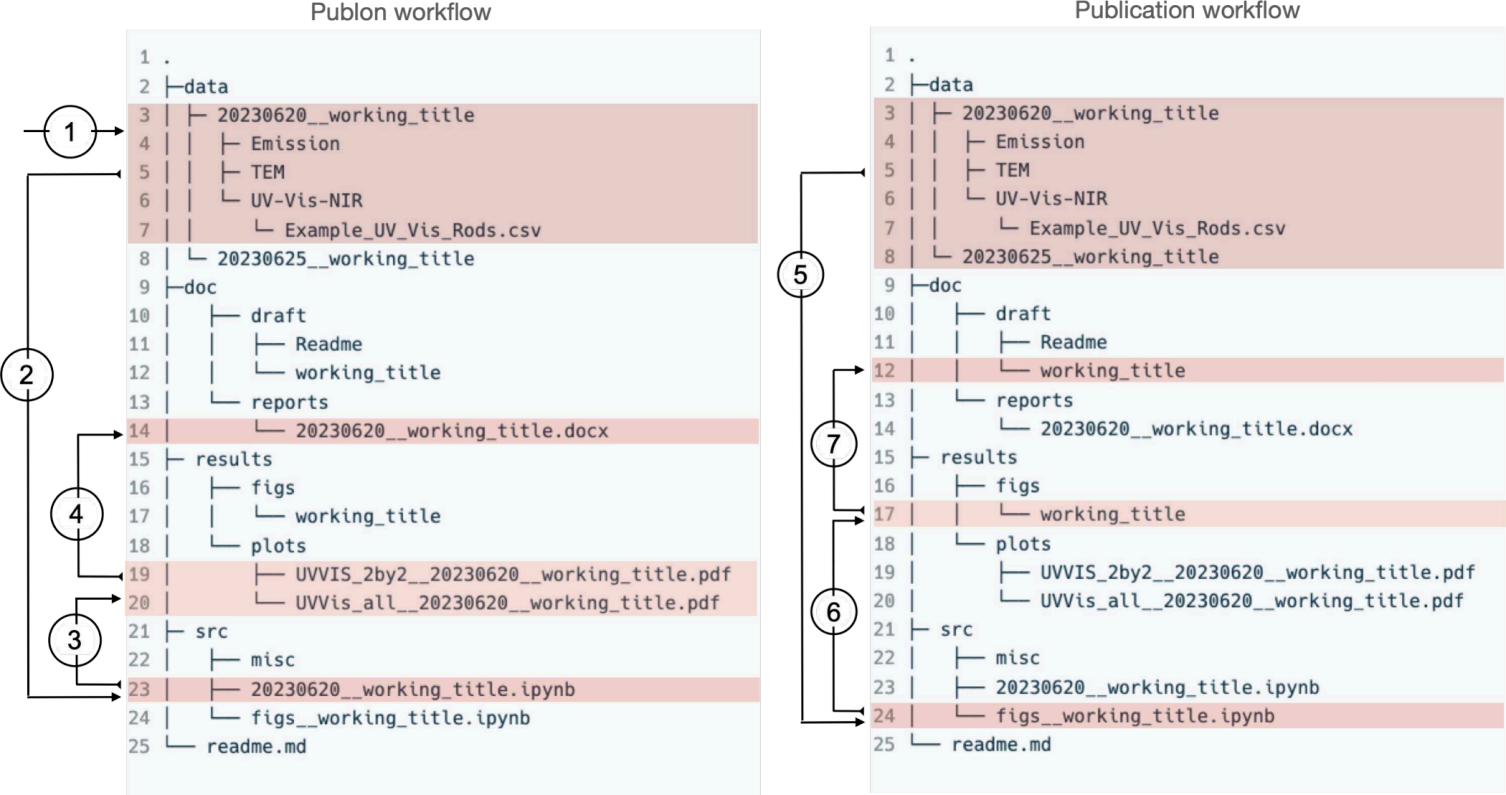
Workflow of data processing. (left) publon. The raw data are imported in the ‘data’ folder (1), that is used by a script in the ‘src’ folder (2) to produce corresponding plots that are saved in the ‘results/plots’ folder (3). These, finally, are incorporated into weekly reports (4). (right) Workflow for publication. The raw data are processed by a script in the ‘src’ folder (5) to produce the corresponding figures that are saved in the ‘results/figures’ folder (6). Final figures are incorporated into draft (7).

### Data Analysis

This is an essential step since it requires critical thinking, considering the current working hypothesis (publon) and higher-level hypothesis (project level). Python programming language is convenient for data analysis (Figure 5, left, step 2). The generated bare plots are stored without stylization (preferably in PDF or PNG file format) in the ‘results/plots’ folder. (Figure 5 left, step 3). Again, attention should be paid to file naming. The name of the plot file contains three domains: ID of plot, ID of publon, and ID of project. The date and working title are the same as those in the raw data and Python script folders. Finally, the generated plot files are used to prepare weekly reports (required time: 3–6 h).

### Reporting

Writing weekly or biweekly reports is vital since all generated data without reporting do not exist. The report should be a self-explaining document, delivering enough information to justify the importance of performed experiments and facilitate understanding the results without going to previous reports (Figure 5, left, step 4). It should contain all components of a typical paper: the main question, stated hypothesis, description of experiments, observations (supported by visual representation), and conclusions. Finally, a paragraph on the next steps can serve as a starting point for the following publon (required time: 3 h).

The accumulation of reports facilitates the progression toward publication. The initial draft of a manuscript is often derived directly from the content contained within publons documents. The critical task involves the creation of figures. The open-source Python programming language is a convenient tool for generating figures. Its transparent approach involves sharing both the data and scripts with the community. This practice ensures reproducibility and allows others to replicate the figures generated in a given project. The process of generation of figures is analogous to plot generation, with the distinction that data are sourced from various publons folders. To produce a figure, a new Jupyter notebook is created, dedicated to generating figures intended for publication (see Figure 5, right, step 5). Separate Jupyter notebooks may serve as figures in the supporting information. The resulting figures are saved in the directory ‘results/figs/working_title’ (see Figure 5, right, step 6). It is important to note that figures generated using Python are typically bare plots containing only essential elements such as axes, labels, and data points. To enhance their clarity, further stylization is needed using specialized software, such as Keynote, PowerPoint, Illustrator, Photoshop, Gimp, or Pixelmator, among others. The native files (e.g., *.key, *.ppt, *.psd) are stored in the ‘results/figs/working_title‘ directory for future editing. It is common to produce between 10 and 25 iterations of each figure to reach a publishable material. The final step involves integrating all figures into the draft manuscript. Abundant captions accompany the figures, explaining their data significance and their contribution to addressing the central research question (see Figure 5, right, step 7).

## Concluding Remarks

The protocol outlined above is designed to offer a shared workflow that can help alleviate the challenges of acquiring and creating new knowledge. It aims to streamline the process, make the pursuit of knowledge manageable and rewarding, and align to some extent with FAIR principles. Also, it is essential to acknowledge the inherent difficulties. There are no AI-based fixes for producing valuable insights, and the notion of effortless productivity remains elusive. It is through this rigorous and energy-intensive journey that we have the opportunity to offer something meaningful. Finally, this paper is open-ended and intended to evolve alongside the ever-changing landscape of tools and methodologies in the academic landscape. Your input is highly valued, and I encourage you to share your suggestions and ideas for enhancing the workflow. Through collaborative work, we can design a standardized protocol based on the publon approach as a tool in nanoscience.
